# Salivary Gland Sonography in Patients with Primary Fibromyalgia—A Pilot Study

**DOI:** 10.3390/life14081043

**Published:** 2024-08-22

**Authors:** Ching-Tsai Lin, Der-Yuan Chen, Yi-Hsing Chen, Chien-Chen Lai, Kuo-Tung Tang

**Affiliations:** 1 Division of Allergy, Immunology and Rheumatology, Taichung Veterans General Hospital, Taichung 407, Taiwan; 2 Faculty of Medicine, National Yang-Ming University, Taipei 112, Taiwan; 3 Rheumatology and Immunology Center, China Medical University Hospital, Taichung 404, Taiwan; 4 College of Medicine, China Medical University, Taichung 404, Taiwan; 5 College of Medicine, National Chung Hsing University, Taichung 402, Taiwan; 6 Ph.D. Program in Translational Medicine, National Chung Hsing University, Taichung 402, Taiwan; lailai@dragon.nchu.edu.tw; 7 Institute of Molecular Biology, National Chung Hsing University, Taichung 402, Taiwan; 8 Graduate Institute of Chinese Medical Science, China Medical University, Taichung 404, Taiwan

**Keywords:** dry eye syndromes, fibromyalgia, Sjogren’s syndrome, ultrasonography, xerostomia

## Abstract

Primary Sjogren’s syndrome (pSS) is often concomitant with fibromyalgia (FM). Salivary gland sonography aids in the diagnosis of pSS. We aimed to discover, in primary FM patients, the presence of pSS in undiagnosed patients through salivary gland sonography. We prospectively recruited 42 primary FM patients. FM symptoms were evaluated based on the revised Fibromyalgia Impact Questionnaire (FIQR). Salivary gland sonography was performed. Patients with positive findings underwent salivary gland biopsy. Comparisons were undertaken using the Mann–Whitney U tests and Chi-squared test. In primary FM patients, the prevalence of dry eye was 83%, and dry mouth was 90%. The salivary gland sonographic score did not differ between patients with and without dry eye/mouth. One patient with a positive finding at salivary gland sonography had a positive result of salivary gland biopsy. In the other four patients who received salivary gland biopsy, despite negative findings in salivary gland sonography, only one had a positive result of salivary gland biopsy. To be noted, scores evaluated by salivary gland sonography were negatively associated with levels of pain (rho = −0.360, *p*= 0.023) and levels of sleep quality (rho = −0.447, *p* = 0.004). Our pilot study demonstrated the potential of salivary gland biopsy in detecting undiagnosed pSS in primary FM patients.

## 1. Introduction

Fibromyalgia (FM) is a debilitating disorder characterized by chronic, widespread pain. It is often accompanied by sleep disturbances, fatigue, and cognitive dysfunction. Epidemiological studies have estimated that FM affects approximately 2% of the general population [1]. Frequently, FM coexists with rheumatic and infectious diseases, complicating its management due to these comorbidities [2,3].

Among patients with primary Sjogren’s syndrome (pSS), the prevalence of FM is around 50% [4,5]. Conversely, the prevalence of pSS in FM patients was reported to be 7%, with the majority being diagnosed via salivary gland biopsy rather than the presence of anti-SSA/B autoantibodies [6]. In a nationwide cohort, the incidence rates of Sjogren’s syndrome were 3.39 and 1.24 per 10,000 person-years in patients with and without FM, respectively [7]. Although salivary gland biopsy is crucial for diagnosis, it is invasive and has low patient acceptance. Moreover, symptoms such as dry eye and dry mouth are common in FM patients [8,9,10]. Thus, distinguishing between primary FM and FM concomitant with pSS is challenging, especially when some pSS patients can only be confirmed through biopsy without detectable autoantibodies [11]. Recent advancements have shown that salivary gland sonography can predict biopsy results, thereby aiding in the diagnosis of pSS [12,13,14]. However, its potential to differentiate primary FM from FM with concomitant pSS remains unexplored. Furthermore, the impact of sicca symptoms on disease manifestations in FM patients has not been thoroughly investigated.

We hypothesize that salivary gland sonography can identify undiagnosed pSS in primary FM patients. Therefore, we conducted a pilot study to screen for pSS in primary FM patients using salivary gland sonography.

## 2. Materials and Methods

### 2.1. Study Participants

We enrolled 42 consecutive primary FM adult outpatients (5 male, 37 female) from the Division of Allergy, Immunology and Rheumatology at Taichung Veterans General Hospital between January 2017 and March 2023. FM was classified according to the 1990 criteria of the American College of Rheumatology (ACR) [15] for 33 patients and according to the 2016 revised version of the 2010/2011 ACR criteria for FM [16] for nine patients. In our center, we first diagnosed FM patients with the stricter 1990 ACR criteria. If the 1990 ACR criteria were not fulfilled and the clinical suspicion remained, we further used the 2016 ACR criteria to make the diagnosis. All patients had negative findings for anti-SSA/B antibodies, which were examined at least once. Patients with concomitant autoimmune diseases were excluded. Our study complied with the Declaration of Helsinki and was approved by the Institutional Review Board of Taichung Veterans General Hospital (IRB TCVGH NO: CE16247B and CE19272B). All patients were recruited after providing written informed consent.

### 2.2. Laboratory Examinations

Serum levels of antinuclear antibodies (ANA) were detected with indirect immunofluorescence using a Hep-2 cell line (Medical & Biological Laboratories, Nagoya, Japan), with positivity set at 1:160. Rheumatoid factor (RF) was examined using nephelometry (Beckman Coulter, Brea, CA, USA). Erythrocyte sedimentation rate (ESR) was determined using the SRS 20/II autoanalyzer (Greiner Bio-One GmbH, Kremsmünster, Austria) with the Westergren method. The normal ranges were 0–10 mm/h for males and 0–20 mm/h for females. High-sensitivity C-reactive protein (hsCRP) was evaluated using immunoturbidimetry (Wako Pure Chemical Industries, Ltd., Osaka, Japan), with a normal range of ≤0.3 mg/dL.

### 2.3. Fibromyalgia Impact Questionnaire

A Chinese version of the Revised Fibromyalgia Impact Questionnaire (FIQR) was used to evaluate disease severity in FM patients [17]. This version was validated previously in 103 Han Chinese patients, with a Cronbach’s α of 0.95 (Whei-Mei Shih, personal communication, 2018 [18]). The maximum FIQR score is 100. The level of pain, energy, sleep quality, depression, memory problems, and anxiety were evaluated using the FIQR, with each answer based on an 11-point numeric rating scale (0 to 10).

Each patient also completed another questionnaire comprising the 2016 revised version of the 2010/2011 ACR criteria for FM [16]. The questionnaire consists of two parts: (a) the widespread pain index (WPI) and (b) the symptom severity (SS) score. The WPI and SS scores were summed to obtain a fibromyalgianess score, representing the severity of FM symptoms. The fibromyalgianess score ranges from 0 to 31 but has not been validated in Chinese patients.

### 2.4. Sicca Symptoms

Patients completed the questionnaire to provide information on their sicca symptoms (i.e., dry eye and dry mouth) prior to using FM medications based on the 2002 criteria of the American-European Consensus Group (AECG) [19]. Ocular symptoms included dry eye, a sensation of sand or gravel in the eyes, or the use of tear substitutes for at least 3 months. If the patient experienced any of these ocular symptoms, the patient was considered to have dry eyes. Oral symptoms included dry mouth, swollen salivary glands, or drinking liquids to aid in swallowing foods for at least 3 months. If the patient experienced any of these oral symptoms, the patient was considered to have dry mouth.

### 2.5. Salivary Gland Sonography

Salivary gland sonography was performed by an experienced and certified rheumatologist (CT Lin) using a scanner (GE Logic 9, Chicago, IL, USA). Grayscale images were obtained with a 15 MHz linear array transducer. The scoring system was based on the evaluation of structural changes in the salivary glands (bilateral parotid and submandibular glands) [20]. Echogenicity, delineation of glandular borders, homogeneity, hypoechoic areas, and hyperechoic foci of the four salivary glands were semi-quantitatively assessed on a four-point scale ranging from 0 to 3. The agreement between the score and salivary gland biopsy result has been shown to be strong, with the score used to diagnose pSS at a cut-off level of 15 [13].

### 2.6. Salivary Gland Biopsy

**Figure 1:**
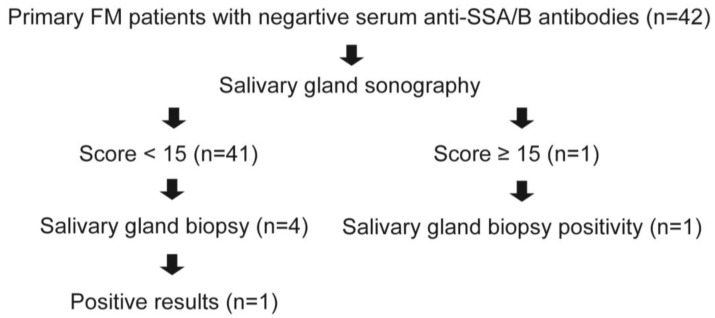
The study algorithm.

For patients with a sonographic score of >14, a labial minor salivary gland biopsy was performed [21]. Additionally, four patients with a sonographic score ≤ 14 also underwent labial salivary gland biopsies after a discussion with their rheumatologists (Figure 1). Patients were classified as having pSS based on the 2016 ACR/European League Against Rheumatism (EULAR) criteria [22].

### 2.7. Statistical Analysis

Values were presented in the median and the interquartile range. Inter-group comparisons were performed using the Mann–Whitney U tests and Chi-squared test. Finally, Spearman’s rank correlation coefficients were used to determine the relationships between clinical parameters and sonographic scores of salivary glands. All statistical analyses were performed using SPSS 18.0 software (IBM Inc., Armonk, NY, USA).

## 3. Results

### 3.1. Demographic Characteristics

Participants’ demographic characteristics are shown in Table 1. Their median age was 44 years, the median disease duration was 5 years, the median fibromyalgianess was 23, and the median FIQR score was 53. Their median level of pain was 7. Half of the patients were taking pregabalin and tramadol.

### 3.2. Features Suggestive of Autoimmunity

Among these primary FM patients, ANA positivity was found in nine (21%), RF positivity in two (5%), and positivity of anti-thyroid antibodies (either anti-thyroglobulin or anti-thyroid peroxidase antibodies) in nine (21%). Elevated inflammatory markers were also observed: ESR was elevated in three (7%), and CRP was elevated in five (12%).

### 3.3. Characteristics of Primary FM Patients with Sicca Symptoms

Among the 42 primary FM patients, 35 (83%) had dry eye symptoms, and 38 (90%) had dry mouth symptoms. A total of 26 (62%) patients had both dry eye and dry mouth symptoms. As shown in Table 2, the characteristics of FM patients were similar regardless of dry eye symptoms. FM patients with dry mouth had fewer tender points and lower levels of pain compared with those without dry mouth. Notably, FM patients with either dry eye or dry mouth had a similar proportion of positive autoantibodies and elevated inflammatory markers compared with those without these symptoms.

### 3.4. Salivary Gland Sonography

**Figure 2:**
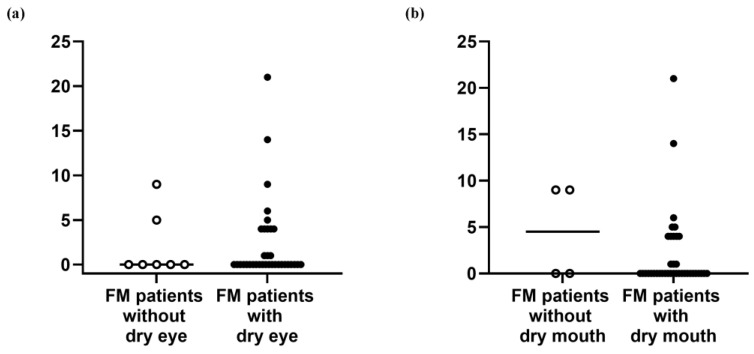
The scores of salivary gland sonography between (**a**) fibromyalgia patients with and without dry eye and (**b**) fibromyalgia patients with and without dry mouth. FM, fibromyalgia.

Sonographic scores of primary FM patients were similar regardless of dry eye/mouth symptoms (Figure 2). One patient who had a score of 21 underwent salivary gland biopsy and had a focus score of ≥1. Additionally, one of the other four patients who underwent salivary gland biopsy also had a focus score of ≥1. These two patients had dry eye, dry mouth, and Schirmer’s test results of ≤5 mm/5 min in at least one eye. They fulfilled the classification criteria of pSS based on the 2016 ACR/EULAR criteria [22].

### 3.5. Correlation Analyses

**Figure 3:**
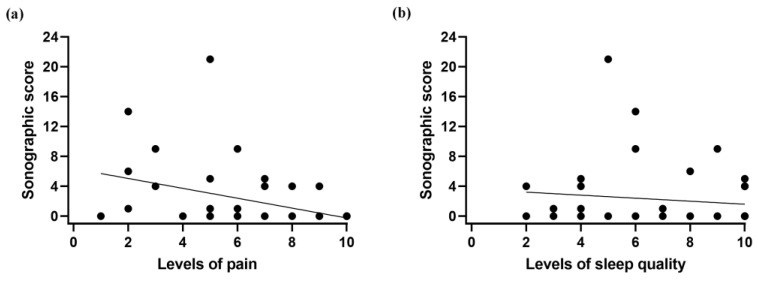
Scatter plots demonstrating correlations between scores evaluated by salivary gland sonography and (**a**) levels of pain and (**b**) levels of sleep quality in fibromyalgia patients.

As shown in Figure 3, salivary gland sonography results were negatively correlated with levels of pain (rho = −0.360, *p* = 0.023) and with levels of sleep quality (rho = −0.447, *p* = 0.004).

## 4. Discussion

The prevalence of sicca symptoms is remarkably high in primary FM patients, leading to a potential underdiagnosis of concomitant pSS. Our pilot study indicates that salivary gland sonography may aid in diagnosing pSS in primary FM patients. A larger-scale study is required to examine the feasibility of the proposed screening algorithm.

The prevalence of sicca symptoms has been reported to range from 6 to 71% in the literature, significantly higher than the general population [9]. However, previous studies did not exclude FM patients, including pSS. In our cohort of primary FM patients, the prevalence of dry eye and dry mouth was 83% and 90%, respectively, after an inquiry about these symptoms. Notably, our FM patients did not have anti-SSA/B autoantibodies at baseline, and some concomitant pSS patients had already been excluded. Furthermore, the proportion of medications used was similar in FM patients regardless of dry eye or dry mouth symptoms, except for a lower proportion of amitriptyline users with dry eye symptoms compared with those without (17% vs. 57%, *p* = 0.023). This underscores the significance of sicca symptoms in the clinical care of primary FM patients, as these symptoms are easily overlooked by physicians. Regarding their impact, sicca symptoms in FM patients were not associated with higher disease severity, such as FIQR score and fibromyalgianess. Conversely, FM patients with dry mouth had fewer tender points and lower levels of pain compared with those without dry mouth.

A higher prevalence of FM has been reported in pSS patients, ranging from 6.9% to 55% [23]. In contrast, the prevalence of pSS in FM patients is less well-known. In an early study, 72 FM patients underwent Schirmer’s test screening, and 28 (38%) showed abnormal results [6]. Among these patients, five (7%) were classified as probable pSS, confirmed by positive salivary gland biopsy results, and two of these patients were positive for anti-SSA/B antibodies. In another cohort of 185 FM patients who screened for autoantibodies, nine (5%) were positive for classical autoantibodies for pSS, including anti-SSA/B antibodies, ANAs, and RF [24]. Gau et al. demonstrated that FM patients had a two-fold higher risk of developing pSS in a claims database [7]. In our cohort of 42 primary FM patients, excluding those with positive anti-SSA/B antibodies, we identified two (5%) patients diagnosed with pSS based on salivary gland biopsy. Consistent with the literature, our results indicate that a small proportion of primary FM patients may still have undiagnosed pSS. Identifying these patients through clinical evaluation is crucial for better FM patient care, as management strategies differ. Additionally, we found that 40% of these primary FM patients had autoantibodies such as RF, ANAs, and anti-thyroid antibodies. These proportions cannot be ignored. Furthermore, seven (17%) of the primary FM patients had elevated inflammation markers like ESR or CRP. Long-term follow-up of these FM patients may provide insight into the clinical significance of these autoantibodies, as previous studies have suggested that FM could be an early sign of developing autoimmune diseases [25].

**Figure 4:**
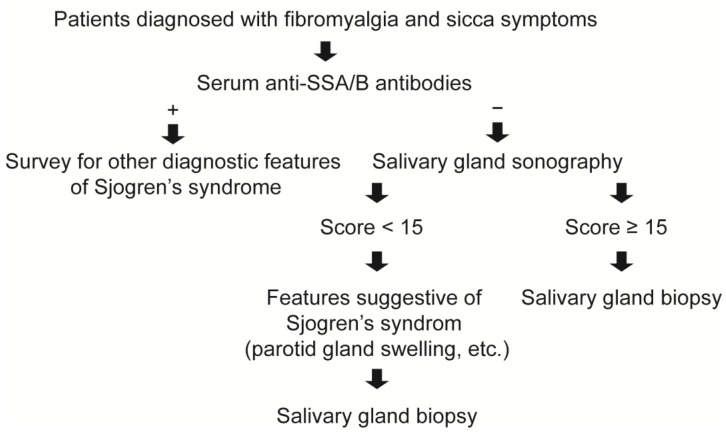
The proposed screening algorithm for Sjogren’s syndrome in fibromyalgia patients.

Salivary gland sonography has been extensively investigated as an aid in the diagnosis of pSS [26]. In a study of 103 suspected pSS patients, the scoring by salivary gland sonography showed a good agreement with the results of parotid (83%) and labial (79%) gland biopsies [13]. When compared to different classification criteria, this scoring demonstrated a sensitivity of 67% to 77% and a specificity of 92% to 94% for diagnosing pSS. The combination of negative salivary gland sonography and the absence of anti-SSA antibodies can highly exclude the classification of pSS. Our preliminary findings align with these results despite a lack of universal salivary gland biopsies for all 42 primary FM patients. One patient with a positive salivary gland sonography result also had a positive salivary gland biopsy result, whereas only one (25%) out of four patients with positive salivary gland sonography had a positive salivary gland biopsy result. We proposed a screening algorithm for pSS in FM patients (Figure 4). Additionally, we did not find a difference in sonographic scores between patients with and without dry eye or dry mouth symptoms. This suggests that the underlying cause of sicca symptoms in primary FM patients is likely unrelated to the inflammatory process. Further studies are needed to explore factors responsible for sicca symptoms in primary FM patients. Interestingly, we found a negative correlation between salivary gland sonography scores and levels of pain and sleep quality. This finding is consistent with our clinical observation that FM patients with dry eye had fewer tender points and lower levels of pain than those without dry eye.

There are some other points that should be mentioned. First, pSS is linked to other neurological disorders, such as Parkinson’s disease, that could lead to dry eye and dry mouth symptoms [27,28,29]. Similarly, salivary gland sonography may help in such a diagnostic challenge, and further studies are needed. Second, there is no distinguishing feature in salivary gland sonography between pSS, sarcoidosis, and amyloidosis [30]. If this is a concern, a salivary gland biopsy is required to make a definite diagnosis. Third, in dentistry, intraoral sonography has been applied to image salivary glands. Its utility in the diagnosis of pSS could be explored in the future [31,32].

Our study had several limitations. First, the sample size is small, and our results need confirmation from a larger cohort. Second, not all patients were screened with salivary gland biopsy, raising questions about the utility of salivary gland sonography. Third, the lack of a control group precludes comparison between primary FM patients and the general population. Fourth, the effect of medications on patients’ sicca symptoms cannot be totally excluded. Fifth, our institution is a tertiary center, and the enrolled patients may have more severe symptoms, such as sicca symptoms.

## 5. Conclusions

In conclusion, our pilot study showed a high prevalence of sicca symptoms in primary FM patients. Facing such a diagnostic challenge, we provided preliminary evidence on the potential utility of salivary gland sonography in detecting undiagnosed pSS in FM patients. In addition, salivary gland sonography scores were negatively associated with levels of pain and sleep quality. Our results need validation in other cohorts.

## Figures and Tables

**Table 1:** Demographic data of all FM patients.

	FM Patients (*n* = 42)
Age, median (IQR) (years)	44 (39, 53)
Female sex, *n* (%)	37 (88%)
Disease duration, median (IQR) (years)	5 (3, 10)
Fibromyalgianess, median (IQR)	23 (20, 26)
Tender points, median (IQR)	13 (11, 16)
ANA positivity, *n* (%)	9 (21%)
RF positivity, *n* (%)	2 (5%)
Positivity of anti-thyroid antibodies	9 (21%)
Elevated ESR, *n* (%)	3 (7%)
Elevated hsCRP, *n* (%)	5 (12%)
FIQR, median (IQR)	53 (31, 63)
Levels of pain, median (IQR)	7 (5, 8)
Levels of energy, median (IQR)	6 (5, 7)
Levels of sleep quality, median (IQR)	8 (5, 9)
Levels of depression, median (IQR)	5 (3, 7)
Levels of memory problems, median (IQR)	6 (4, 8)
Levels of anxiety, median (IQR)	7 (5, 9)
Medications, *n* (%)	
Pregabalin	22 (52%)
Tramadol 37.5 mg/acetaminophen 325 mg	15 (36%)
Tramadol	21 (50%)
Amitriptyline	10 (24%)

ANA, antinuclear antibody; hsCRP, high-sensitivity C-reactive protein; ESR, erythrocyte sedimentation rate; FIQR, the revised fibromyalgia impact questionnaire; FM, fibromyalgia; IQR, interquartile range; RF, rheumatoid factor.

**Table 2:** Characteristics of FM patients with and without dry eye/mouth symptoms.

	FM Patients with Dry Eye (*n* = 35)	FM Patients without Dry Eye (*n* = 7)	FM Patients with Dry Mouth (*n* = 38)	FM Patients without Dry Mouth (*n* = 4)
Age, median (IQR) (years)	44 (39, 53)	44 (40, 56)	43.5 (39, 53)	50 (42, 63)
Female sex, *n* (%)	30 (86%)	7 (100%)	33 (87%)	4 (100%)
Disease duration, median (IQR) (years)	5 (3, 11)	5 (3, 6)	5 (3, 11)	5 (3.5, 6)
Fibromyalgianess, median (IQR)	23 (19, 27)	22 (20, 25)	23 (20, 26)	23 (18, 25)
Teder points, median (IQR)	15 (11, 17)	11 (6, 13)	15 (11, 17)	10 (5, 11.5) *
ANA positivity, *n* (%)	8 (23%)	1 (14%)	8 (21%)	1 (25%)
RF positivity, *n* (%)	2 (6%)	0 (0%)	2 (5%)	0 (0%)
Thyroid autoimmunity, *n* (%)	8 (23%)	1 (14%)	9 (24%)	0 (0%)
Elevated ESR, *n* (%)	3 (9%)	0 (0%)	3 (8%)	0 (0%)
Elevated hsCRP, *n* (%)	4 (12%)	1 (14%)	4 (11%)	1 (25%)
FIQR, median (IQR)	58 (30, 63)	46 (41, 67)	54 (36, 63)	39 (23, 64)
Levels of pain, median (IQR)	7 (5, 8)	6 (6, 7)	7 (5, 8)	4.5 (2, 6) *
Levels of energy, median (IQR)	6 (5, 8)	6 (3, 7)	6 (5, 8)	3 (0.5, 6)
Levels of sleep quality, median (IQR)	8 (5, 10)	8 (4, 9)	8 (5, 10)	7 (4, 8.5)
Levels of depression, median (IQR)	5 (3, 7)	5 (2, 7)	5 (3, 7)	5 (3.5, 6)
Levels of memory problems, median (IQR)	6 (5, 8)	5 (4, 8)	6 (4, 8)	6.5 (4, 8)
Levels of anxiety, median (IQR)	7 (5, 9)	8 (6, 9)	7 (5, 9)	6 (5, 7)

ANA, antinuclear antibody; hsCRP, high-sensitivity C-reactive protein; ESR, erythrocyte sedimentation rate; FIQR, the revised fibromyalgia impact questionnaire; FM, fibromyalgia; IQR, interquartile range; RF, rheumatoid factor. * *p* < 0.05.

## Data Availability

The data that support the findings of this study are available from the corresponding author.
